# Does Women’s Empowerment in Agriculture Matter for Children’s Health Status? Insights from Northern Ghana

**DOI:** 10.1007/s11205-016-1328-z

**Published:** 2016-04-18

**Authors:** Yacob A. Zereyesus, Vincent Amanor-Boadu, Kara L. Ross, Aleksan Shanoyan

**Affiliations:** 1 Department of Agricultural Economics, Kansas State University, 307B Waters Hall, Manhattan, KS 66506 USA; 2 Department of Agricultural Economics, Kansas State University, 306 Waters Hall, Manhattan, KS 66506 USA; 3 Department of Agricultural Economics, Kansas State University, 305D Waters Hall, Manhattan, KS 66506 USA

**Keywords:** Women’s empowerment in agriculture, Latent variable, Height-for-age, Weight-for-height, MIMIC

## Abstract

Given that women in rural communities in developing countries are responsible for the nutrition and health-related decisions affecting children in their care, their empowerment may influence the health status of their children. The association between women’s empowerment, measured by using a recently developed Women’s Empowerment in Agriculture Index, and children’s health status is examined for a sample of households in Northern Ghana applying a Multiple Indicators Multiple Causes (MIMIC) model. The MIMIC approach is used to link multiple indicator variables with multiple independent variables through a “single underlying” latent variable. Height-for-age and weight-for-height z-scores are used as indicators of the underlying children’s health status and women’s empowerment in agriculture and control variables are used as the multiple independent variables. Our results show that neither the composite empowerment score used to capture women’s empowerment in agriculture nor its decomposed components are statistically significant in their association with the latent children’s health status. However, the associations between children’s health status and control variables such as mother’s education, child’s age, household’s hunger scale and residence locale are statistically significant. Results also confirm the existence of the ‘single underlying’ common latent variable. Of the two health status indicators, height-for-age scores and weight-for- height scores, the former exhibited a relatively stronger association with the latent health status. While promoting women’s empowerment to enhance their ability to make strategic life choices, it is important to carefully consider how the achievement of these objectives will impact the women’s well-being and the well-being of the children in their care.

## Introduction

Research shows that healthy children perform well in school and achieve higher educational attainment because they are more likely to be attentive (Brown and Pollitt 1996; Rivera et al. 1995). Educational attainment has been shown to correlate with incomes (Psacharopoulos and Patrinos 2004) as well as mental and physical state (Duflo 2000). Thus, children’s health status is deemed an important factor in their ultimate human capital capacity and economic productivity (Alderman et al. 2006; Behrman 1996; Grantham-McGregor et al. 1999; Victora et al. 2008).

Women, as the traditional caregivers of children (especially in rural communities in developing countries) are particularly responsible for the nutrition and other health-related decisions affecting children in their care (Saaka et al. 2009). The choices available to women caregivers are not independent of their own capabilities, resources and freedoms to make independent decisions (Boateng et al. 2014). Interest in women’s ability to make independent choices, framed as women’s empowerment, has become an increasingly important subject in economic development circles over the past few decades (Doan and Bisharat 1990; Narayan 2002; Alsop et al. 2006). Although women’s empowerment is a complex and multidimensional concept, the general consensus is that women’s empowerment refers to “the enhancement of women’s ability to make strategic life choices.” (Malhotra and Schuler 2005, p. 84). It is believed that empowering women can contribute to achieving development goals such as poverty reduction and gains in human capital formation such as improved health status for women and education. These positive externalities can also lead to increases in agricultural productivity, income growth, and improvements in child health (Smith et al. 2003; Quisumbing and Maluccio 2003; Pitt and Khandker 1998). For example, in Bangladesh, Pitt and Khandker (1998) showed that credit provided to women led to higher household consumption expenditures and to more schooling for girls. Quisumbing (2003) also reported that women’s bargaining power resulted in increased expenditure shares on education in Bangladesh.

Given women’s role as primary caregivers of children, it is reasonable to assume that their empowerment would influence the health status of their children. Previous studies that have examined the relationship between women’s empowerment on children’s health status have found that women’s bargaining power affects the intrahousehold resource allocation pattern supporting children’s health and development (Haddad and Hoddinott 1994; Desai and Johnson 2005; Heaton and Forste 2008; Radebe et al. 1996; Quisumbing and Maluccio 2003; Glick and Sahn 1998; Lundberg et al. 1997; Bégin et al. 1999; Engle 1993). In their study in Mazowe district of Zimbabwe, Radebe et al. (1996) found that children whose mothers had low decision making ability were more likely to suffer from malnutrition. Bégin et al. (1999) examined how caregiver characteristics influence children’s nutritional and health status and found differing results depending on the children’s age. Maternal influence on what foods their children were given was associated with higher height-for-age z-scores for younger children, but not for older children. In Cote d’Ivoire, Haddad and Hoddinott (1994) found that an increase in the wife’s share of household’s income was associated with better nutritional status of children. Carlson et al.’s (2015) summary of the relationship between various measurements of women’s autonomy and child nutritional status suggests that improvements in women’s autonomy could be associated with better children’s health status. However, the majority of the previous studies have based their analysis on a single measure of children’s health status, while children’s health and well-being have been accepted as a multidimensional concept encompassing physical, mental, psychological, material deprivation, and social aspects (Yarcheski et al. 1994; Lee 2009). Although a single indicator could provide useful insights on a specific dimension of children’s well-being, examination of multiple indicators could provide a better appreciation of their health status, especially if the indicators may be correlated to each other and there is possible measurement error in the individual indicators (Williams 2005). Consequently, studies based on multidimensional concept of children’s health status, and utilizing multiple indicators for these dimensions could make valuable extensions to the existing literature in this area. The current study specifically focuses on the physical aspect of children’s well-being as a reflection of the overall status of good physical health and nourishment (Nussbaum 1999). The child’s height-for-age and weight-for-height measurements are used as the observable indicators of physical health, which are manifestations of the latent, error free, variable for child’s health status (Di Tommaso 2007).

This paper aims to explore the empirical association between women’s empowerment in agriculture and the health status of children in their care. To achieve this objective, a Multiple Indicators Multiple Causes (MIMIC) model is employed to link multiple indicator variables with multiple independent variables through a “single underlying” latent variable. This MIMIC model allows for controlling possible measurement error in the individual indicator variables (Williams 2005). Previous studies using different forms of the composite women’s empowerment index have reported mixed results. Desai and Johnson (2005), for example, found no statistically significant association between women’s autonomy and children’s height-for-age in Benin, Uganda, Zimbabwe, Egypt, Nepal, Colombia, Nicaragua and Peru. However, they reported significant positive association in India and Mali, and significant negative association in Malawi. Heaton and Forste (2008) also found no association between women’s autonomy and children’s height-for-age in Colombia, Peru, Nicaragua and Bolivia, but a negative association in Haiti. A special strength of this paper arises from the use of a recently developed aggregate measure known as the Women’s Empowerment in Agriculture Index (WEAI) developed to examine women’s empowerment in broader rural settings, whether they are farmers, agricultural or non-agricultural wage workers, or engaged in non-farm businesses (Alkire et al. 2013). A distinct feature of the WEAI is that it incorporates multiple crucial aspects of empowerment such as women’s time allocation, control over resources and social engagement which makes it superior to previous measures of women’s empowerment which mostly relied on a single variable or composite index (Carlson et al. 2015). The new index is also useful in identifying key aspects of disempowerment that need to be strengthened and to track their progress over time (Alkire et al. 2013). Thus, while promoting women’s empowerment as a vehicle to enhance their ability to make strategic life choices, the results of this paper based on the new WEAI provide important insights for policy makers and development agencies to carefully consider how the achievement of such objectives will impact the women’s wellbeing and the well-being of their children.

The remainder of the paper is organized as follows: the data and methods sections present the nature and sources of the data and the development of the model, respectively. The subsequent results section presents the main empirical findings and discussions of the study. Conclusions and policy implications are summed up in the last section.

## Data

This study uses data from 2012 population-based survey conducted in Ghana. Ghana is a West African country, with an estimated population in 2012 of about 24 million. Although it has been performing very well against the Millennium Development Goals of the United Nations (United Nations 2000), Ghana’s performance is uneven across its administrative regions (Osei-Assibey and Grey 2013). For example, the three northernmost regions were all found to be lagging behind the national average on poverty reduction goals. As a result of this uneven progress, the majority of development agencies, including the U.S. Agency for International Development (USAID), are now focusing their development efforts in the northern part of the country.

The 2012 population-based survey was conducted in the area above Ghana’s Latitude 8°N covering the administrative regions of Brong Ahafo, Northern, Upper East and Upper West, but excluding the areas falling in Volta Region. The total population in the study area was estimated at about 5.2 million in 2012, a little over 20 percent of Ghana’s total population.

The survey was commissioned by the USAID with an objective of providing baseline estimates for monitoring and evaluation of its Feed the Future intervention activities in Northern Ghana. The total sample of the survey included 4410 households and nearly 25,000 men, women and children. However, for the purpose of this research a sub-sample is selected consisting of 1393 of women aged less than 50 years (reproductive age women) who had children below 5 years of age in their care. Survey weights were used to ensure the sample estimates are representative of the entire population of interest. In addition to demographic and socio-economic information, the survey collected data on children and women’s anthropometry, and data to facilitate the estimation of the Women’s Empowerment in Agriculture Index (WEAI), household hunger scale and women’s dietary diversity scores.

The current research was approved for compliance with federal, state or local rules, regulations and guidelines by the appropriate Research Compliance Office, the Committee on Research Involving Human Subjects which serves as the Institutional Review Board (IRB). Informed consent was also obtained from all individual participants included in the survey.

The anthropometric indicators of height-for-age and weight-for-height are developed to represent the underlying children’s health status. These two observable anthropometric indicators which are considered endogenous to the model represent the unobservable children’s health status outcome variable. It is noted that well-nourished children 10 years or younger, regardless of their country and ethnic backgrounds, have similar height and weight distribution and growth rates (Cogill 2003). This allows for the development of a reference population that can be used to facilitate children’s anthropometric measurement comparisons. It is therefore necessary to standardize the anthropometric measures when using them in any analysis (United Nations 2006). The standardization involves developing the z-score, *Z*_*ij*_, for each child, *j*, in the sample and each anthropometric indicator, *i*, such that:

1 $$Z_{ij} = {{(V_{ij} - V_{Mi} )} \mathord{\left/ {\vphantom {{(V_{ij} - V_{Mi} )} {\sigma_{Mi} }}} \right. \kern-0pt} {\sigma_{Mi} }}$$

where *V*_*ij*_ is the observed value for the *i*th indicator of the *j*th child, and $V_{Mi}$ and $\sigma_{Mi}$ are the median and the standard deviation of the *i*th indicator in the reference population. When $Z_{ij}$ for any child is more than 2 standard deviations below *V*_*Mi*_, then that child is considered stunted or wasted for *i* equals height-for-age or weight-for-height, respectively.

Two alternative models are utilized in this study; the difference between them is that one uses the composite index used to capture women’s empowerment in agriculture and the other uses the principal components of the WEAI as explanatory variables. Both models also incorporate demographic and socio-economic characteristics of the children and their parents as exogenous variables as well as household hunger and the diversity in participating women’s diets.

WEAI has two weighted sub-indexes: the Five Domains of Empowerment (5DE); and the Gender Parity Index (GPI) (Alkire et al. 2013). The 5DE and the GPI indexes have 90 percent and 10 percent of the weights in the derivation of the WEAI, respectively. The GPI measures a woman’s empowerment relative to her male counterpart in household. The 5DE, on the other hand, assesses the extent of woman’s empowerment in decision-making and control across five domains of empowerment examined under the WEAI: production, resources, income, leadership, and time. The production domain assesses a woman’s sole or joint decision-making authority in agricultural production, whether crop or livestock farming or fisheries. The resources domain assesses ownership, access to, and decision-making power over productive resources such as land, livestock, agricultural equipment, credit, and consumer durables while the income domain assesses whether sole or joint control over income and expenditures. The leadership domain explores a woman’s membership in economic or social groups and her comfort speaking in public while the time domain evaluates her satisfaction with the distribution of her time between work and leisure. Each of the 5DE comprises one, two or three components, giving rise to a total of ten components; each of them allocated a weight the sum of which is unity. Table 1 provides the criteria used to determine the extent of empowerment for each of the ten components. The weighted sum of stated short fall in the ten components for each woman provides a measure of the inadequacy count (CI). A woman is considered not yet empowered, or disempowered, if her CI is at least 20 percent (Alkire et al. 2013). Recall that we explore the effect of both the composite and decomposed forms of the WEAI. In its composite form, we treat the CI as a continuous variable. However, in the decomposed form, we treat each component as a dummy variable.

**Table 1:** The adequacy criteria and weights used for the indicators in the five domains of empowerment in agriculture. *Source*: Alkire et al. (2013)

Domain	Indicator	Adequacy criteria	Weight
Production	Input in productive decisions	A woman is adequate if she participates or feels she has input in at least two types of decisions	1/10
	Autonomy in production	A woman has adequate achievement if her actions are motivated more by her values as opposed to her fear of disproval or feelings of coercion	1/10
Resources	Ownership of assets	A woman is adequate if she has joint or sole ownership of at least one major asset	1/15
	Purchase, sale, or transfer of assets	On assets owned by a household, a woman is adequate if she is involved in the decisions to buy, sell, or transfer assets	1/15
	Access to and decisions on credit	An adequate woman belongs to a household that has access to credit and when decisions on credit are made, she has input in at least one decision regarding at least one source credit	1/15
Income	Control over use of income	A woman is adequate if she has some input (or perceived input) on income decisions provided that she participated in the income generating activity	1/5
Leadership	Group member	A woman is considered adequate if she is a member of at least one group from a wide range of economic and social groups	1/10
	Speaking in public	A woman is deemed adequate if she is comfortable speaking in public in at least one context	1/10
Time	Leisure time	A woman has adequate leisure time if she does not express any level of dissatisfaction with the amount of leisure time available	1/10
	Work burden	A woman is considered to have an excessive workload and thus, inadequate if she worked more than 10.5 h in the previous 24 h	1/10

Table 2 presents the definition and summary statistics of the WEAI variables and other variables used in the models. The mean scores on the WEAI show the proportion of women who are not yet empowered by each of the 10 indicators within each of the five domains of empowerment. More than 70 percent of the women have inadequate achievement in the purchase, sale, or transfer of assets; and in access to and decisions on credit. In terms of the time domain, 46 percent of the women do not have a manageable work load, whereas only 14 percent of the women have expressed dissatisfaction with their leisure time. A little over 30 percent of the women do not have any group membership and 27 percent of them do not feel comfortable speaking in public. The average inadequacy score of disempowered women is 0.36, indicating that despite the high disempowerment among these women; they experience adequate achievement in 64 percent of the empowerment domains.

**Table 2:** Summary statistics of the principal variables used in the analysis

Variable	Description	Mean	SD
*Women’s empowerment in agriculture variables*			
Inadequacy count		0.36	0.18
Input in productive decisions	1 = Inadequate; 0 = Adequate	0.34	0.47
Autonomy in production	1 = Inadequate; 0 = Adequate	0.32	0.47
Ownership of assets	1 = Inadequate; 0 = Adequate	0.43	0.5
Purchase, sale, or transfer of assets	1 = Inadequate; 0 = Adequate	0.75	0.43
Access to and decisions on credit	1 = Inadequate; 0 = Adequate	0.78	0.42
Control over use of income	1 = Inadequate; 0 = Adequate	0.21	0.41
Group member	1 = Inadequate; 0 = Adequate	0.32	0.46
Speaking in public	1 = Inadequate; 0 = Adequate	0.27	0.45
Work burden	1 = Inadequate; 0 = Adequate	0.46	0.5
Leisure time	1 = Inadequate; 0 = Adequate	0.14	0.35
*Demographic and socioeconomic variables*			
Age of child	Months	29.93	16.56
Gender of child	1 = Male; 0 = Female	0.49	0.50
Education of mother	1 = Some formal educational training; 0 = No education	0.05	0.21
Age of mother	Years	33.17	9.43
Women’s dietary diversity score	Women’s Dietary Diversity	4.13	1.46
Education of father	1 = Some formal educational training; 0 = No education	0.13	0.33
Household hunger scale	1 = Moderate to severe hunger; 0 = Little to no hunger	0.36	0.48
Income deciles		4.70	2.56
*Household and location characteristics*			
Household size		8.06	4.27
Safe drinking water	1 = Household drinking water safe; 0 = otherwise	0.68	0.47
Locale	1 = Urban; 0 = Rural	0.18	0.38
*Children’s health status variables*			
Height-for-age	z-scores	−1.41	1.99
Weight-for-height	z-scores	−0.24	1.66

The remaining control variables are the same in both models. They include women’s dietary diversity score, household hunger scale and the socio-economic characteristics of mother’s age, both father’s and mother’s education, household income, residence locale, child’s gender and age. Due to the high risk of safe water on health, we also include access to portable drinking water as a dummy exogenous variable. It may be expected that the exogenous variables will exhibit multicollinearity. However, the Variance Inflation Factor (VIF) values for all the variables were below 2, suggesting that these variables were not collinear.

The Women’s Dietary Diversity Score (WDDS) is developed using a count of nine food groups consumed over 24 h preceding the interview (Kennedy et al. 2011). There are three categories of the score: (1) Low—consuming foods from no more than three food groups; (2) Medium—consuming foods from four to five food groups; and (3) High—consuming foods from more than five of the food groups. The Household Hunger Scale (HHS) is a simple indicator for tracking household hunger in food insecure areas (Ballard et al. 2011). The HHS is estimated from answers to a series of questions about food accessibility and the frequency of food insecurity over a four-week recall period (Ballard et al. 2011). The food insecurity of a household is considered severe when the HHS is between 4 and 6 and moderate when it between 2 and 3. The household is considered to have little or no hunger when its HHS is between 0 and 1. The study sample includes children with a relatively equal representation of male and female children, a mean age of 30 months, living in primarily rural areas. The average age of the mother was 33 years with a dietary diversity score of 4.13, implying that the average mother consumes 4 out of the 9 food groups. The prevalence rate of households experiencing moderate to severe hunger in the 4 weeks prior to the survey is 36 percent. The average z-scores for both the children’s height-for-age and weight-for-height measurements imply that the children in the sample on average have scores below the median reference population values.

## Methods

**Fig. 1:**
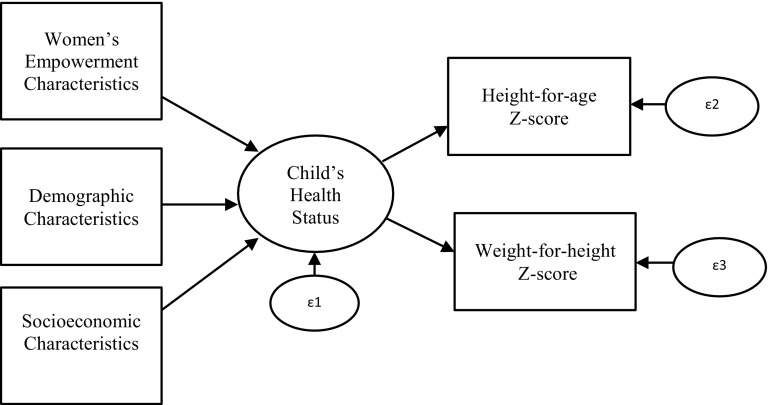
*Path diagram* of the MIMIC model of children’s health status in Northern Ghana. The *path diagram* shows that there is a direct influence from the exogenous variables to the latent variable (children’s health status) and from the latent variable to the anthropometric indicators. The exogenous variables are grouped into socio-economic characteristics, demographic characteristics, and women’s empowerment characteristics. Following convention, a coefficient in the diagram is fixed to one to identify the model and provide a scale for the latent health variable

The methods used in this paper rely on a Multiple Indicators Multiple Causes (MIMIC) model. The MIMIC model is a distinct specification of the Structural Equation Modelling (SEM) approach useful when multiple dependent variables need to be tied together with multiple independent variables through a “single underlying” variable. The structure of the model can be represented by the path diagram shown in Fig. 1 (Bollen 1989). The observed height-for-age and weight-for-height z-scores represent the underlying latent children’s health status. The path diagram shows that there is a direct influence from the women’s empowerment and other control variables to the latent variable, children’s health status, which has height-for-age and weight-for-height scores as indicators. Because these two variables are not independent of each other makes the research problem a good candidate for MIMIC modeling. Using multiple indicators is also beneficial because it reduces the effect of measurement error of any individual variable on the accuracy of the overall results (Williams 2005). Di Tommaso (2007), for example, employed the MIMIC model to study the effect of parents’ literacy and the child’s gender, among other variables, on children’s wellbeing in India. She defined wellbeing to encompass physical health, imagination and thought, and leisure and play activities. Mabsout (2011) also used the MIMIC model to explore women’s health as indicated by their body mass index and anemia status, reporting that women’s health can be improved by changing household decision-making patterns.

The measures on height-for-age and weight-for-height determine the health status of a child at any particular point in time. These indicators are continuous variables and represent infinite possibilities of the health status of the child. When the height-for-age (weight-for-height) z-score for any child is more than two standard deviations below the median score of a reference population, the child will be considered as stunted (wasted). Stunting indicates chronic malnutrition resulting from prolonged periods of inadequate nutrition or recurrent or current illness. It may, thus, be interpreted as an indication of poor conditions in the child’s environment. Stunting affects the children’s cognitive development, which may limit their production capacity. Wasting, on the other hand, is typically an indication of acute under-nutrition resulting from insufficient food intake and/or a highly infectious disease. The effect of wasting on the immune system can be severe, leading to increased severity and duration of existing diseases as well as susceptibility to infectious diseases (WHO 2010).

At any given time, a child can express one of these three health status states as measured by height-for-age and weight-for-height scores: (1) a child can neither be stunted nor wasted; (2) a child can either be stunted or wasted; or (3) a child can be both stunted and wasted. In the latter case, a stunted child who has been suffering from chronic malnutrition as a result of prolonged periods of inadequate nutrition may also become wasted if the child happens to be exposed to short term malnutrition or to a high infectious disease incidence. Therefore, we may represent the underlying latent health status of the child by the particular scores of these two indicators at any given time. We may develop a structural relationship between a vector, $X = (x_{1} \ldots x_{n} )^{\prime}$, of observable causal variables and the latent children’s health status, Y* (Joreskog and Goldberger 1975; Spanos 1984). Equation 2 describes this structural relationship between Y* and the observable exogenous variables as:

2 $$Y^{*} = \alpha^{\prime} X + \varepsilon$$

where ε is the error term, assumed to have a zero mean and a unity standard deviation, and $\alpha = (\alpha_{1} \ldots \alpha_{n} )^{\prime}$ is a vector of the parameters to be estimated. The latent children’s health status above is assumed to determine the observable health status indicators, Y, giving rise to the measurement component of the SEM as follows:

3 $$Y = \beta Y^{*} + \upsilon$$

where $Y = (y_{1} \ldots y_{m} )^{\prime}$ represents a vector of observable endogenous variables, $\beta = (\beta_{1} \ldots \beta_{n} )^{\prime}$ is a vector of parameters to be estimated, and $\upsilon = (\upsilon_{1} \ldots \upsilon_{m} )^{\prime}$ is a vector of mutually independent error terms. It is assumed that $E(\varepsilon \upsilon^{\prime} ) = 0$, $E(\varepsilon^{2} ) = \sigma^{2}$, and $E(\upsilon \upsilon^{\prime} ) = \Theta^{2}$, with $\Theta$ being an $m\, \times m$ diagonal matrix.

The MIMIC model, which is the reduced form of Eqs. 2 and 3 presents the observable health status indicators, Y, as a function of the observable exogenous variables, X, suggesting that:

4 $$Y = \pi^{\prime} X + \nu$$

where $$\pi = \alpha \beta^{\prime}$$ and $$v = \beta \varepsilon + \upsilon^{\prime}$$

The MIMIC model is identified when there are at least two observable indicators and at least one exogenous variable, and one of the factor loadings of the observable indicators is set to unity to provide a scale for the latent variable. The research problem meets this threshold requirement for the MIMIC model to be identified, making its application appropriate. The MIMIC model is estimated using a maximum likelihood method and we set the factor loadings of the height-for-age indicator to unity to provide a scale for the latent variable.

The exogenous variables in our model have different units, making any comparison of their estimated parameters uninformative. Bollen (1989) provides solution to this problem, suggesting standardization of the estimated parameters to eliminate their units, and in doing so, make them comparable. This is akin to economists using elasticities to determine the relative importance of the contributions of variables. The problem with the elasticity metaphor is that the contribution approaches infinity as the point of the estimation approaches zero. Bollen’s (1989) solution avoids this problem by standardizing using standard deviations. From Eqs. 2 and 3 above, the standardized parameter estimates, $\hat{\alpha }_{ij}^{s}$ and $\hat{\beta }_{ij}^{s}$, are therefore defined as:

$$\hat{\alpha }_{ij}^{s} = \hat{\alpha }_{ij} \left( {\frac{{\hat{\sigma }_{jj} }}{{\hat{\sigma }_{ii} }}} \right) \quad {\text{and}}\quad \hat{\beta }_{ij}^{s} = \hat{\beta }_{ij} \left( {\frac{{\hat{\sigma }_{jj} }}{{\hat{\sigma }_{ii} }}} \right)$$

where $i$ is the dependent variable, $j$ is the explanatory variable, $\hat{\sigma }_{ii}$ and $\hat{\sigma }_{jj}$ are the model-predicted standard deviations of the *i*th and *j*th variables, respectively. The standardized parameter estimates show the change in standard deviation units of the dependent variable resulting from a one standard deviation change in an explanatory variable, *ceteris paribus*.

## Results

Table 3 shows the results of the two models: the composite WEAI using respondents’ inadequacy count as a continuous variable; and the decomposed WEAI using the 5DE components as binary variables. When probability weights are applied to make the estimated results representative of the entire population, goodness of fit indicators are given by the Standardized Root Mean Squared Residuals (SRMR).The SRMR scores for both specifications were less than 0.05, indicating good fit of the models. The association between the composite inadequacy count (CI) index used to capture women’s empowerment in agriculture and children’s health status is not statistically significant in Northern Ghana. Similarly, the associations between the decomposed WEAI components and children’s health status are not statistically significant. These results are in line with previous results in which they found no statistically significant association between women’s autonomy and children’s height-for-age in Benin, Uganda, Zimbabwe, Egypt, Nepal, Colombia, Nicaragua and Peru (Desai and Johnson’s 2005) and Colombia, Peru, Nicaragua and Bolivia (Heaton and Forste 2008). Previous studies using different forms of the composite women’s empowerment index have reported varying results. For example, Smith et al. (2003) studied the associated between women’s relative decision-making power within a household and children’s health status in 36 countries. They found that higher women’s power was positively associated with child’s health status as measured by weight-for-height, weight-for-age and height-for-age, but this relationship was not consistent across all 36 countries. In particular, Ghana showed a negative relationship between women’s power and children’s weight-for-age.

**Table 3:** Regression results of the structural MIMIC model

	Specification 1		Specification 2	
	Standardized coefficient	SE	Standardized coefficient	SE
Input in productive decisions			0.030	0.036
Autonomy in production			0.042	0.040
Ownership of assets			0.075	0.040
Purchase, sale, or transfer of assets			−0.050	0.040
Access to and decisions on credit			0.016	0.045
Control over use of income			−0.038	0.041
Group member			−0.019	0.035
Speaking in public			0.053	0.038
Work burden			−0.060	0.033
Leisure time			−0.001	0.026
Inadequacy count	0.005	0.032		
Child’s age	−0.217***	0.050	−0.231***	0.053
Child’s gender	−0.037	0.035	−0.046	0.039
Mother’s education	0.062**	0.030	0.074**	0.033
Mother’s age	0.057	0.033	0.063	0.035
Women’s dietary diversity score	0.050	0.032	0.054	0.031
Father’s education	−0.001	0.030	−0.002	0.032
Household hunger scale	0.105***	0.033	0.101***	0.034
Income deciles	0.006	0.043	0.011	0.049
Household size	0.110	0.058	0.105	0.065
Safe drinking water	0.056	0.035	0.055	0.040
Locale	0.098**	0.040	0.107**	0.042

**, *** Significance of standardized coefficients at the 95 and 99 % confidence levels, respectively. The Standardized Root Mean Squared Residual (SRMR) for both specifications are 0.009 and the R^2^ for specification 1 and 2 are 0.10 and 0.12, respectively

To the best of our knowledge, no research has been conducted on assessing the association between the individual components of the 5DE and children’s health status. What our results show is that none of the 10 components exhibited a statistically significant association with children’s health status in the study area. However some of the control variables are associated with children’s health status. In both specifications, child’s age and household’s hunger scale have a statistically significant association with children’s health status at the 1 percent level while mother’s education and locale, defined as whether the family lived in a rural or urban area are determined to have a statistically significant association with children’s health status at the 5 percent level. The signs on both parameter estimates are as expected, i.e., the higher a child’s age, the lower the associated height-for-age score, and the more educated the child’s mother, the higher the child’s height-for-age score. This result is in agreement with previous results that suggest mother’s education is positively correlated with the children’s well-being in Uganda (Shariff and Ahn 1995). Children living in urban areas are also associated with having higher height-to-age scores. The associations between children’s health status and family income, father’s education, and drinking water are not statistically significant. The results also provide additional information on the estimated standardized coefficients. For example, the marginal analysis of the standardized coefficients show that, holding other factors constant, a one standard deviation shift in child’s age would shift child’s health status by 0.22 and 0.23 standard deviations in Model Specification 1 and 2, respectively.

**Fig. 2:**
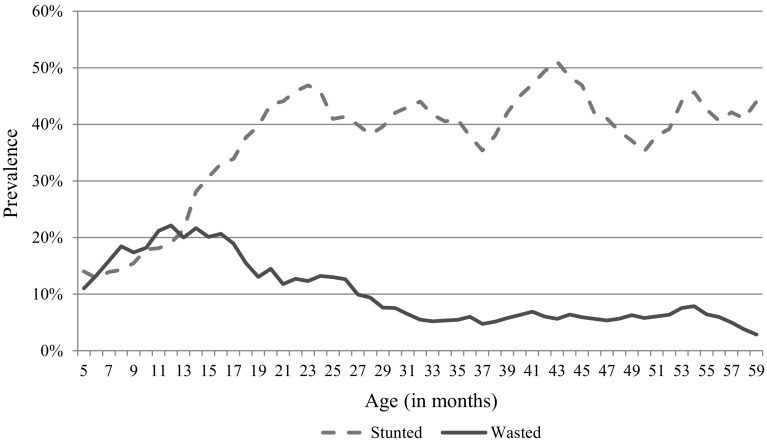
6 months moving averages of the prevalence of stunting and wasting in children less than 5 years of age in Northern Ghana *Source*: authors’ calculations

The results from the measurement models also reveal the magnitude and strength of the association between the health status variable and its constituent indicators (Table 4). The statistically significant coefficients of the height-for-age and weight-for-height scores confirm the existence of the underlying ‘single’ common latent variable. Furthermore, the factor loadings indicate that the underlying common health status variable is more associated with the height-for-age than with the weight-for-height scores. Thus, a one standard deviation change in the latent health status variable is associated with 0.94 standard deviation change in the height-for-age score and about 0.36 standard deviation change in the weight-for-height score. The different signs on the estimated coefficients of the two indicators of children’s health status reveal the negative correlation between them. To further gain insight into such observed relationship, the trends of the prevalence of stunting and wasting during the first 5 years of the children’s age are provided in Fig. 2. The figure shows the 6 months moving averages of both indicators of health status. Although the average prevalence of both the stunting and wasting in children increase very rapidly in the first 1 year of the children’s life, only stunting continues to increase until the end of the second year. Additionally, while the trend in the average of the prevalence of wasting declines with the age of the children; the prevalence of stunting seems to persist at higher levels even as the children approach 5 years of age. This may be an indication that while wasting related problems may be addressed effectively with time, stunting related problem may be more challenging to address.

**Table 4:** Regression results of the measurement MIMIC model

	Specification 1		Specification 2	
	Standardized coefficient	SE	Standardized coefficient	SE
Height-for-age	0.990***	0.164	0.940***	0.185
Weight-for-height	−0.336***	0.071	−0.359***	0.090

*** Significance of standardized coefficients at the 99 % confidence level. The R^2^ values for specifications 1 and 2 are 0.98 and 0.88, respectively

## Implications and Conclusions

Women’s empowerment has become an increasingly important factor in economic development. There has, therefore, been an increasing interest to examine its role in influencing development indicators, such as children’s health status. The motivation for this research was, thus, to contribute to the exploration of the effect of women’s empowerment on children’s health status by looking at both the composite and decomposed forms of Alkire et al.’s (2013) WEAI. Defining health status by two indicators—height-for-age and weight-for-height z-scores—the study employed the Multiple Indicator Multiple Causes (MIMIC) model to assess the effect of WEAI by controlling the effect of demographic and other socio-economic variables on children’s health status. Our results showed that empowerment domains of WEAI, either as a composite variable or decomposed into its 10 components, are not significant in explaining children’s health status in Northern Ghana. Previous studies using different formats of the composite women’s empowerment index have also reported mixed results. However, it should be noted that the current results pertain specifically if the empowerment domains used to construct the WEAI in the context of farming families matter to children’s health status in Northern Ghana.

The control variables that showed statistically significant associations with children’s health status at 5 % or lower were mother’s education, child’s age, household hunger scale and locale. The findings of the current research support the importance of educating women as an instrument in economic development, a point that has received more attention for longer than women’s empowerment (King and Hill 1998) and the possibility that educating girls may directly address any gaps in women’s empowerment (Medel-Anonuevo and Bochynek 1993).

Ghana’s Ministry of Women’s and Children’s Affairs (MOWAC) has a mandate to promote the welfare of women and children, their survival, development and protection (ROG 2004). In meeting MOWAC’s objectives of promoting women’s equal access to, and control over economically significant resources and benefits, it is important to carefully consider how the achievement of these objectives will impact the women’s wellbeing and the well-being of the children in their care.

Finally, the study’s results show that the variability in children’s health status is associated more with children’s height-for-age z-scores than with weight-for-height z-scores. As an indicator of long term chronic child malnutrition, it has been shown that childhood height-for-age challenges, exhibited through stunting for example, tend to persist through to adulthood (Grantham-McGregor et al. 2007). Interventions to address child and toddler health issues must, therefore, pay particular attention to this to help avert (or at least minimize) the long-term implications of this childhood health problem on overall human capital development and economic growth.
